# Harnessing pathogen stress-hormone sensing for living medicines

**DOI:** 10.1371/journal.pbio.3003940

**Published:** 2026-08-26

**Authors:** Weston R. Whitaker

**Affiliations:** Departments of Veterinary and Biomedical Sciences and of Biochemistry and Molecular Biology, The Pennsylvania State University, University Park, Pennsylvania, United States of America

## Abstract

Pathogens sense host stress hormones in the gut and use them to regulate motility and virulence. This Primer discusses a new PLOS Biology study which transfers this pathway to Escherichia coli Nissle and redesigns its regulation to drive programmable outputs.

## Teaser

Pathogens sense host stress hormones in the gut and use them to regulate motility and virulence. A new study in *PLOS Biology* transfers this pathway to *Escherichia coli* Nissle and redesigns its regulation to drive programmable outputs.

Pathogens can eavesdrop on the body’s stress response. The catecholamines norepinephrine (NE; also known as noradrenaline) and epinephrine (Epi; also known as adrenaline) reach the intestine through circulation and local release from sympathetic neurons, where gut microbes can further alter their availability through uptake and metabolism [1,2]. Enteric pathogens use these host molecules to regulate growth, motility, and virulence [2,3]. Signals normally described as communication among host tissues can therefore become environmental cues for bacteria. Giving engineered commensals access to the same neural and endocrine signals could create new forms of physiological control, but natural sensing pathways are entwined with the regulatory networks of their original hosts. In this issue of *PLOS Biology*, Srivastava and colleagues show how the adrenergic-sensing QseBC pathway from enterohemorrhagic *Escherichia coli* (EHEC) can be transferred into the clinically relevant probiotic *E. coli* Nissle 1917 and rebuilt as a modular catecholamine-responsive system [4,5].

**Fig 1:**
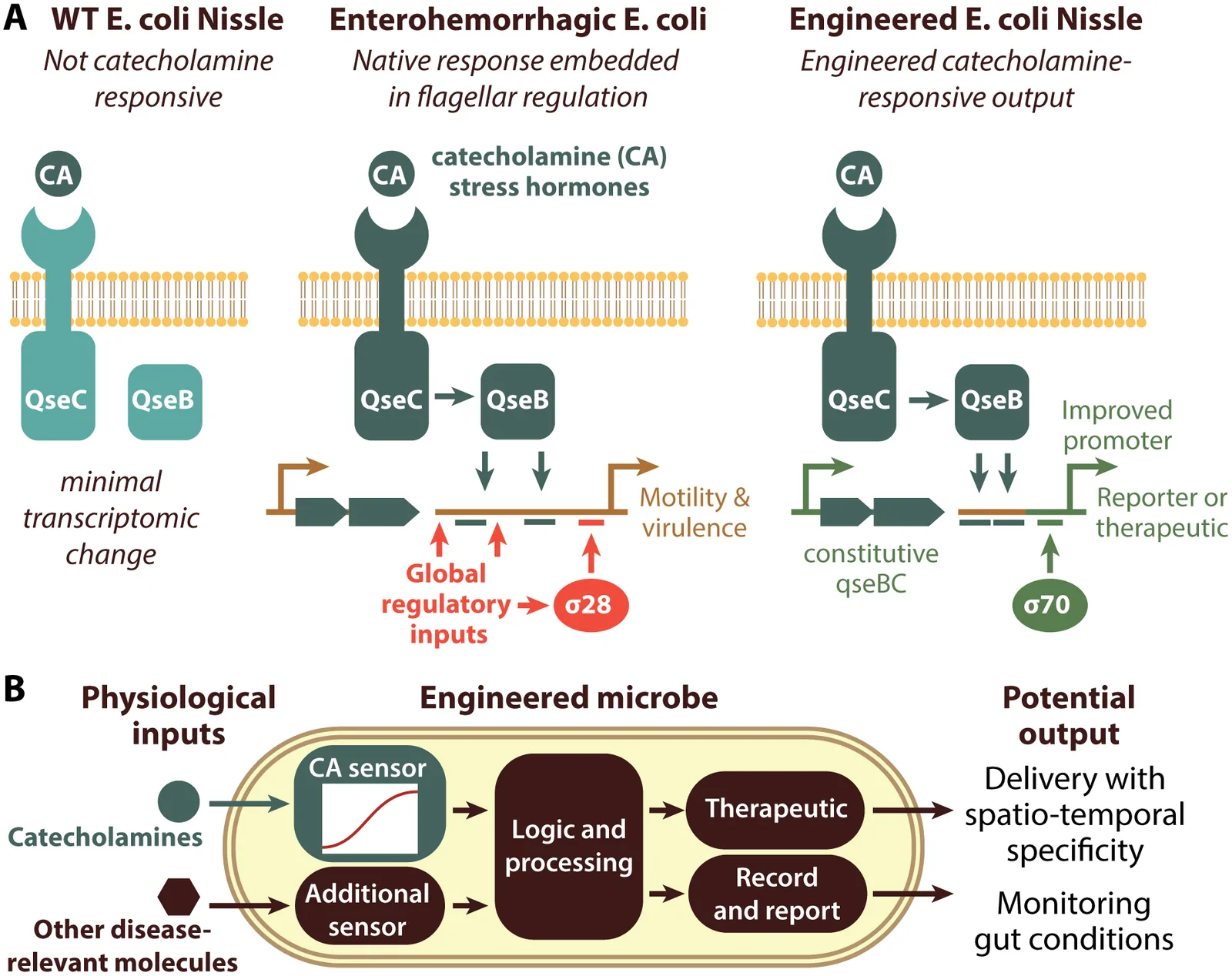
Engineering catecholamine-responsive control in *Escherichia coli* Nissle 1917. **(A)** Wild-type *E. coli* Nissle encodes a QseBC two-component system closely related to that of enterohemorrhagic *E. coli* (EHEC), but showed minimal transcriptional responses to the catecholamines norepinephrine and epinephrine under the conditions tested. In EHEC, catecholamine-responsive QseBC signaling feeds into the native *flh*DC promoter, which is embedded in a broader network of global and flagellar regulation, including an apparent sigma-28-dependent promoter core. Srivastava and colleagues transferred EHEC *qse*BC into *E. coli* Nissle and reconstructed its transcriptional output by tuning constitutive *qse*BC expression, truncating the native promoter, replacing the apparent sigma-28-dependent core with synthetic sigma-70-dependent promoters, and restoring a QseB-associated motif. The resulting circuit retained catecholamine responsiveness while producing a larger and more consistent transcriptional response that could control a reporter or therapeutic cargo, yielding a portable catecholamine-sensing module. **(B)** Future circuit architectures could combine catecholamine-sensing with additional disease-relevant physiological inputs and use amplification, logic, thresholding, or memory to process these signals. Such circuits could couple therapeutic production to particular physiological conditions or generate a retrievable record of signals encountered in the gut. Figure created in Adobe Illustrator.

*E. coli* Nissle already encodes QseBC, a two-component system in which the membrane sensor kinase QseC detects an extracellular signal and phosphorylates its partner response regulator QseB, which in turn alters gene transcription. Its QseB and QseC proteins are ~97% identical, at the amino acid level, to their EHEC counterparts. Transcriptomic analysis showed that NE elicited minimal changes in wild-type Nissle, whereas expression of EHEC QseBC in Nissle produced broader NE-dependent modulation of genes involved in flagellar biogenesis, quorum-sensing, stress adaptation, and biofilm formation [5]. To identify promoters suitable for NE-dependent expression of heterologous proteins, the authors placed several EHEC flagellar promoters upstream of a red fluorescent reporter in Nissle harboring EHEC QseBC. Only the EHEC flhDC promoter showed significant, though weak, NE-dependent activation. The flhDC promoter integrates several regulatory inputs, including global regulators and an apparent sigma-28-dependent core linked to the FliA-FlgM flagellar checkpoint, potentially weakening expression and introducing unwanted regulatory complexity. The authors therefore shortened the promoter to remove upstream sequences and tuned QseBC expression (Fig 1A), improving responsiveness. To further reduce context-dependent variability, they replaced the apparent sigma-28 core with synthetic sigma-70 promoters and restored QseB-binding sites. Their best construct produced ~4-fold induction, compared with less than 1.5-fold induction from the most responsive unmodified promoter. Importantly, this reconstruction separated the output from parts of its native flagellar regulation, simplifying interpretation of sensor output and future engineering.

The study also begins to dissect the sensor itself. Structure-guided mutagenesis identified QseC sensor-domain residues that contribute to signaling. Mutating these residues reduced responsiveness to both catecholamines and the quorum-sensing molecule AI-3. Although these mutations do not yet explain why the highly similar EHEC and Nissle proteins differ in ligand response, they provide a tractable starting point for mapping QseC function. The authors then connected the engineered promoter to secretion of rCRF(9-41), a rat α-helical antagonist of the corticotropin-releasing factor (CRF) receptor. When CRF was co-administered with LPS, pretreatment with purified rCRF(9-41) expressed from the catecholamine-sensing module inhibited the resulting pro-inflammatory cytokine expression in differentiated THP-1 macrophages and reduced the increase in paracellular permeability in Caco-2 cells, showing that the expressed cargo retains biological activity. This work marks an early but concrete step toward therapeutics that couple production to the host signals they sense.

What might such a catecholamine-sensing module enable? Engineered gut bacteria have already been programmed to sense inflammation-associated metabolites, dietary inputs, and other intestinal conditions, and either respond immediately or store a lasting memory of exposure [6,7]. QseBC extends this toolkit to a host neuroendocrine signal that can vary across time and gut location. Unlike a conventional drug with a dosing schedule set outside the body, an engineered bacterium could adjust production according to signals where it resides. This could be useful for diseases that fluctuate over time or are spatially localized. A direct responsive design, such as the one developed by Srivastava and colleagues, could release a therapeutic cargo during catecholamine exposure. A second sensor for inflammation or anatomical location could add context, so that output requires a particular combination of host and local signals. Alternatively, a strain could provide therapy continuously while a memory circuit records catecholamine exposure. Sequencing bacteria recovered from stool could then relate exposure history to treatment response [8] (Fig 1B).

Realizing these designs requires calibrating what QseBC activity means in vivo. A full test will have to demonstrate sensitivity and specificity of the module in the complex molecular environment of the gut. Because the circuit also responds to AI-3, its output may reflect microbial quorum signaling as much as host stress. Additionally, which catecholamines bacteria encounter is not a simple readout of host stress, as these hormones are released by nerves locally within the gut wall, and how much reaches the gut interior in active form is itself shaped by the resident microbes [1,2]. The optimized promoters produced ~4-fold induction, which may suffice to trigger an amplifier or memory circuit but may fall short for directly producing some protein cargos, where greater dynamic range or a higher maximum output could be needed. Beyond the sensor itself, live bacterial therapeutics still face the basic constraints of achieving sufficient therapeutic activity and bacterial abundance while ensuring biocontainment [9,10]. Such measures can themselves alter the cellular state in which the sensor operates. Whether the response stays stable and interpretable across these contexts remains to be shown, though removing the complex native regulatory inputs makes that calibration more tractable.

Despite starting with a challenging, weakly induced, and highly cross-regulated pathway, Srivastava and colleagues provide a practical template for developing sensing modules with improved dynamic range and reduced context dependency. They have demonstrated a strategy that can be applied to other host-sensing pathways to broaden the range of physiological signals available to engineered microbes and support therapeutic strains that both respond to and record conditions encountered in vivo.
